# Patterns of asthma medication use and hospital discharges in New Zealand

**DOI:** 10.1016/j.jacig.2024.100258

**Published:** 2024-04-10

**Authors:** Jonathan Noble, Lee Hatter, Allie Eathorne, Thomas Hills, Orlagh Bean, Pepa Bruce, Mark Weatherall, Richard Beasley

**Affiliations:** a Medical Research Institute of New Zealand, Wellington, New Zealand; b Victoria University of Wellington, Wellington, New Zealand; c University of Otago Wellington, Wellington, New Zealand

**Keywords:** Asthma, dispensing, guidelines, hospital admission, ICS/formoterol, New Zealand

## Abstract

**Background:**

In New Zealand a progressive increase in budesonide/formoterol dispensing, accompanied by a reduction in dispensing of short-acting β_2_-agonists (SABAs), inhaled corticosteroids (ICSs), and other ICS/long-acting β_2_-agonists (ICSs/LABAs), occurred in the 18-month period following publication of the 2020 New Zealand asthma guidelines, which recommended budesonide/formoterol anti-inflammatory reliever therapy.

**Objective:**

Our aim was to investigate more recent trends in asthma medication use and asthma hospital discharges in New Zealand.

**Methods:**

New Zealand national dispensing data for inhalers for the period from January 2010 to December 2022 were reviewed for patients aged 12 years and older. Monthly rates of dispensing of budesonide/formoterol, ICSs, other ICS/LABAs, and SABAs were displayed graphically by locally weighted scatterplot smoother plots. The rates of dispensing and hospital discharge for asthma were compared between the past 6 months for which dispensing data were available (July-December 2022) and the corresponding period from July to December 2019.

**Results:**

There has been a progressive increase in dispensing of budesonide/formoterol since 2019, with a 108% increase between the period from July to December 2019 and the period from July to December 2022 in adolescents and adults. In contrast, there was a reduction in rates of dispensing of other ICS/LABAs, ICSs, and SABAs by 3%, 18%, and 5%, respectively. During this period, there was a 17% reduction in hospital discharges for asthma.

**Conclusion:**

There has been a further widespread uptake of ICS/formoterol reliever and/or maintenance therapy in adolescents and adults with asthma in New Zealand. The changes in prescribing practice have been temporally associated with a reduction in hospital admissions for asthma.

## Introduction

In 2020, the New Zealand adolescent and adult asthma guidelines took the novel approach of recommending a stand-alone stepwise treatment algorithm specifically incorporating budesonide/formoterol reliever therapy as the preferred management approach.^1^ In the 18 months after release of the guidelines, there was a 65% increase in dispensing of budesonide/formoterol for patients aged 12 years or older, together with a reduction in dispensing of short-acting β_2_-agonists (SABAs), inhaled corticosteroids (ICSs), and other ICS/long-acting β_2_-agonists (LABAs), suggesting a widespread transition to budesonide/formoterol maintenance and/or reliever therapy regimens in clinical practice.^2^ Over the same time period, there was a progressive reduction in hospital discharges for asthma; however, there was also a reduction in hospital discharges related to chronic obstructive pulmonary disease (COPD) and pneumonia, likely due to fewer respiratory virus infections as a result of coronavirus disease 2019 (COVID-19) public health measures implemented in New Zealand.^3^^,^^4^

In this report, we extend our enquiry by an additional 12 months to further investigate these temporal patterns of asthma prescribing and outcomes as New Zealand relaxed public health measures in response to the transition of COVID-19 from a pandemic to an endemic infection.^5^

Monthly inhaled medication dispensing data were provided by the New Zealand Pharmaceutical Management Agency (PHARMAC) for January 2010 to December 2022 for (1) SABAs, (2) budesonide/formoterol (the only ICS/formoterol product available in New Zealand), (3) other ICS/LABAs (fluticasone propionate/salmeterol and fluticasone furoate/vilanterol), and (4) ICSs (not as an ICS/LABA [ie, beclomethasone propionate, fluticasone propionate, and budesonide]). The term *dispensing* combines both the initial medication(s) dispensed and any instances of repeat dispensing authorized by the same prescription.^6^ New Zealand has means-tested, government-subsidized primary health care and universal secondary health care, with a prescription fee for inhalers equivalent to about $3 (US) per item in the observation period.

The primary outcome was the rate of dispensing per 100,000 population in those aged 12 years or older (ie, the group to which the 2020 New Zealand adolescent and adult asthma guideline recommendations apply).^1^ In sensitivity analyses, the rates of dispensing in the separate age groups younger than 12 years, 12 to 34 years, 35 to 49 years, 50 to 64 years, and 65 years and older were presented.

Hospital discharge data for the period from January 2013 to December 2022, were provided by Te Whatu Ora Health New Zealand, from the National Minimum Data Set, for the International Classification of Diseases codes for asthma (J45-J46), COPD (J40-J44), and pneumonia (J12-J18).

A locally weighted scatterplot smoother (LOESS) was used to produce a smoothed curve of (1) dispensed medication rates by time and (2) hospital discharge rates by time. The denominator for rate is the estimated New Zealand resident population from the beginning of the first quarter of 2010 to the end of the fourth quarter of 2022.^7^ All population estimates at June 30, 2018, and beyond use the 2018 baseline data, which are based on the 2018 census.

For (1) each medication class and (2) each hospital discharge diagnosis, the relative changes were calculated and expressed as proportions comparing the period from July 2022 to December 2022 (the most recent 6-month period for which medication data were available) with the corresponding 6-month period 3 years earlier (July-December 2019), which was before the New Zealand COVID-19 public health measures were put in place. For the analysis, SAS software, version 9.4 (SAS Institute, Cary, NC), was used.

## Results and discussion

**Fig 1:**
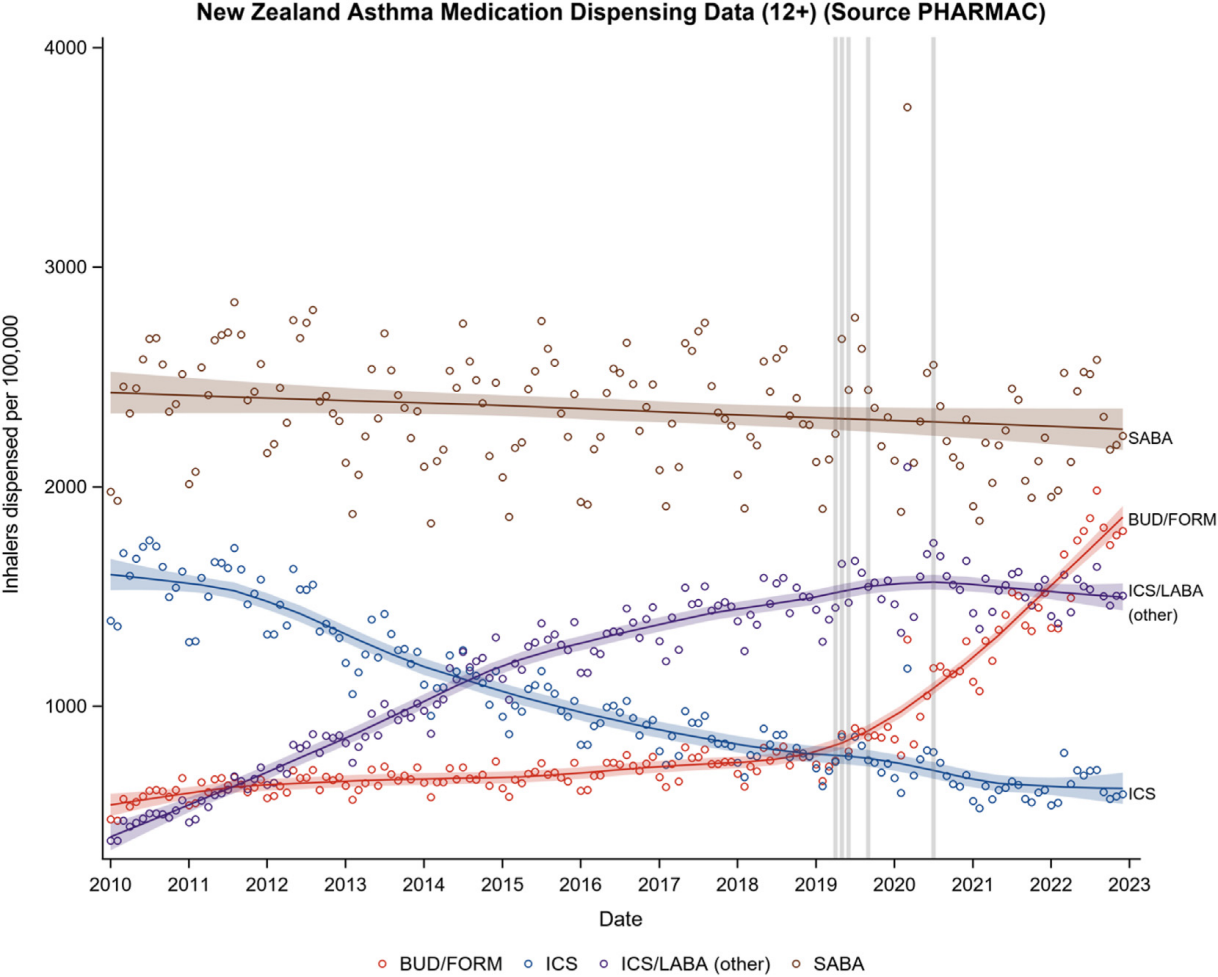
LOESS plot of medication dispensing rates for adolescents and adults (aged ≥12 years) monthly from January 2010 to December 2022. Gray vertical lines indicate the date of publication of the 2019 Global Initiative for Asthma update (April 2019),^8^ publication of the results of the Novel START trial (May 2019),^9^ approval of the indication for prescription of budesonide/formoterol (BUD/FORM) 200/6 μg as a reliever alone in New Zealand (June 2019),^10^ publication of the results of the PRACTICAL trial (September 2019),^11^ and publication of the New Zealand adolescent and adult asthma guidelines (June 2020).^1^ Shaded bands represent the 95% CIs.

In the age 12 and older group, there has been a progressive, marked increase in budesonide/formoterol dispensing since 2019 (Fig 1^8^, ^9^, ^10^, ^11^). In the past 6 months for which data are available (July-December 2022), the rate of dispensing of budesonide/formoterol inhalers was 10,973 per 100,000 compared with 5,283 per 100,000 in the period from July to December 2019 (ie, a 108% relative increase [Table I]).

**Table I:** Difference in the rate of dispensing of inhalers between the period from July to December 2019 and the period from July to December 2022 by medication class in the group of those aged 12 or older

Inhaler type	Inhalers dispensed per 100,000 population			
	Jul-Dec 2019	Jul-Dec 2022	Difference	% Change
Budesonide-formoterol	5283.01	10972.76	5689.75	+107.7
ICS	4625.05	3801.50	–823.55	–17.8
ICS-LABA (other)	9448.23	9144.22	–304.01	–3.2
SABA	14707.03	14007.47	–699.56	–4.8

There was a leveling off in dispensing of other ICSs/LABAs, from around 2020 (Fig 1). The rate of dispensing of other ICS/LABA inhalers in the period from July to December 2022 was 9,144 per 100,000 compared with 9,448 per 100,000 in the period from July to December 2019, representing a relative decrease of 3% (Table I).

Throughout the period of observation, there has been a progressive reduction of ICS dispensing, with no apparent change in the rate in recent years (Fig 1). The rate of dispensing of ICS inhalers in the period from July to December 2022 was 3,802 per 100,000 compared with 4,625 per 100,000 in July to December 2019, representing a relative decrease of 18% (Table I).

There has been a small reduction in SABAs dispensed during the period of observation, with no apparent change in rate in recent years (Fig 1). The rate of dispensing of SABA inhalers in the period from July to December 2022 was 14,007 per 100,000 compared with 14,707 per 100,000 in the period from July to December 2019, representing a relative decrease of 5% (Table I).

The trends in dispensing of inhaled medication differed in a number of respects across the age groups 12 to 34 years, 35 to 49 years, 50 to 64 years, and 65 years and older (see Fig E1 and Table E1 in the Online Repository at www.jaci-global.org).

**Fig 2:**
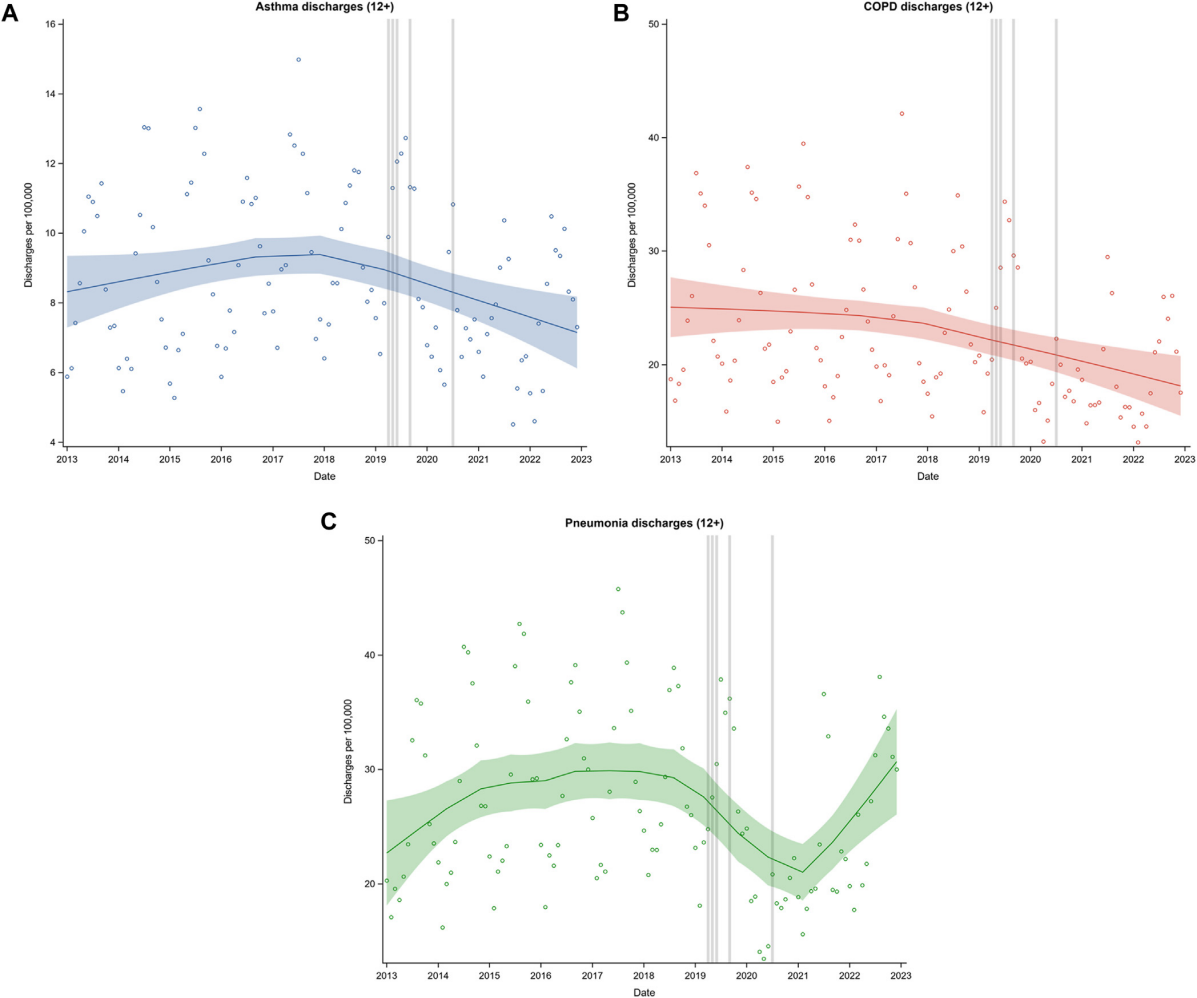
LOESS plot of hospital discharge rates for adolescents and adults (aged >12 years) monthly from January 2013 to December 2022. **A,** Asthma (*blue*). **B,** COPD (*pink*). **C,** Pneumonia (*green*). Gray vertical lines indicate the date of the publication of the 2019 Global Initiative for Asthma update (April 2019),^8^ publication of the results of the Novel START trial (May 2019),^9^ approval of the indication to prescribe budesonide/formoterol 200/6 μg as a reliever alone in New Zealand (June 2019),^10^ publication of the results of the PRACTICAL trial (September 2019),^11^ and publication of the the New Zealand adolescent and adult asthma guidelines (June 2020).^1^ Shaded bands represent the 95% CIs.

In the age group of those aged 12 years or older, there was a steady increase in hospital discharge rates for asthma up until about 2018, followed by a steady decrease (Fig 2, *A*^8^, ^9^, ^10^, ^11^). The rate of hospital asthma discharges was 17% lower in the period from July to December 2022 than in the period from July to December 2019 (Table II). There was a steady decrease in hospital discharge rates for COPD until about 2018, after which there was a more marked decrease (Fig 2, *B***)**. The rate of hospital COPD discharges was 18% lower in the period from July to December 2022 than in the period from July to December 2019 (Table II). There was an increase in hospital pneumonia discharge rates until about 2017 and then a marked reduction until 2021, followed by a marked increase (Fig 2, *C*).

**Table II:** Difference in the rates of hospital admissions for asthma, COPD, and pneumonia between the period from July to December 2019 and the period from July to December 2022 in the group of those aged 12 and older

Diagnosis	Hospital discharges per 100,000 population			
	Jul-Dec 2019	Jul-Dec 2022	Difference	% Change
Asthma	63.63	52.72	–10.91	–17.1
COPD	165.96	136.87	–29.09	–17.5
Pneumonia	193.42	198.69	5.26	2.7

**Fig 3:**
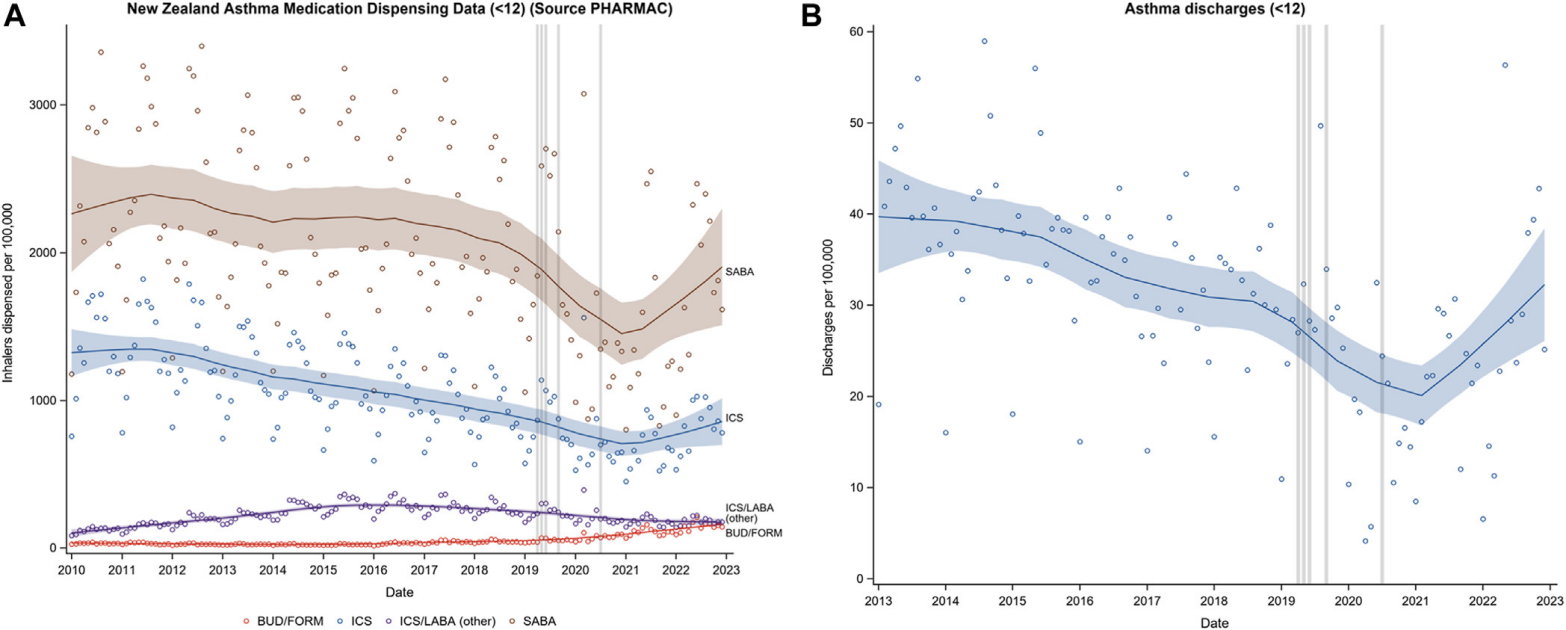
LOESS plots of medication dispensing rates (**A**) and hospital discharge rates for asthma monthly from January 2010 to December 2022 (**B**) for children younger than 12 years, monthly from January 2013 to December 2022. Gray vertical lines indicate the date of the publication of the 2019 Global Initiative for Asthma update (April 2019),^8^ publication of results of the Novel START trial (May 2019),^9^ approval of the indication to prescribe budesonide/formoterol (BUD/FORM) 200/6 μg as a reliever alone in New Zealand (June 2019),^10^ publication of the results of the PRACTICAL trial (September 2019),^11^ and publication of the the New Zealand adolescent and adult asthma guidelines (June 2020).^1^ Shaded bands represent the 95% CIs.

In the age group of those younger than 12 years, the trends in medication dispensing and asthma hospital discharges are shown in Table E1 and Fig 3.^8^, ^9^, ^10^, ^11^

These analyses demonstrate further progressive uptake of budesonide/formoterol maintenance and/or reliever therapy in clinical practice in New Zealand, with more than a 2-fold increase from the period from July to December 2019 to the period from July to December 2022 in adolescents and adults aged 12 years and older. This trend was temporally associated with a 17% reduction in hospital asthma discharges.

Although there has been a leveling off and/or modest reduction in the rates of dispensing of ICSs, other ICSs/LABAs, and SABAs during this period, it is of lesser combined magnitude than the increase in rates dispensing of budesonide/formoterol, suggesting that there is likely to have been some degree of coprescribing of a SABA as a reliever. This interpretation is limited by the lack of information from individual patient prescriptions.

To interrogate the issue of confounding by COPD, we undertook age group sensitivity analyses, which revealed that the proportional increase in budesonide/formoterol dispensing was substantially greater in the group of those aged 12 to 34 years than in the group of those aged 65 years or older, and the proportional decrease in the dispensing of other ICSs/LABAs and SABAs was substantially greater. These observations suggest likely confounding with dispensing of other ICSs/LABAs and SABAs to the substantive population of patients with COPD in the older age group, thereby overrepresenting their use in the broad population across all age groups.

In our previous time trend analyses,^2^ it was difficult to interpret the associated changes in hospital asthma discharges owing to the marked reduction in respiratory virus infections resulting from the strict COVID-19 public health measures implemented in New Zealand.^3^^,^^4^^,^^12^ This limitation was overcome to some degree with this current analysis, which extended the period of observation by another 12 months, when COVID-19 public health measures were eased and respiratory infections were once again widely prevalent in the population.^13^^,^^14^

The point of inflection for the marked, progressive increase in dispensing rates for budesonide/formoterol began during 2019, which was around the time of recommendation of ICS/formoterol as the preferred reliever across the range of asthma severity in the Global Initiative for Asthma 2019 update and,^8^ publication of the results of the New Zealand budesonide/formoterol reliever therapy clinical trials,^9^^,^^11^ and approval of the indication for prescription of budesonide/formoterol 200/6 μg for use in New Zealand as a reliever alone.^10^ The inflection point for the progressive reduction in hospital asthma discharge rates also occurred around this time point. A different pattern was observed with pneumonia, for which rates increased until 2017, decreased from 2019, and then markedly increased again from 2021. Although there are numerous potential confounding factors, these observations provide weak evidence that such a transition in budesonide/formoterol prescribing contributed to the reduced population risk of asthma hospital admission.

This interpretation is also supported by the different time trends in children younger than 12 years, for whom there was a very low baseline level of budesonide/formoterol dispensing and a marked increase in hospital asthma discharges late in 2021-2022 compared with a decrease in the group of those aged 12 years and older during this same period. As viral respiratory tract infections are the predominant cause of severe exacerbations of asthma in both children and adults,^15^^,^^16^ these findings are broadly consistent with the interpretation that the prescribing patterns in the group of those aged 12 years and older have contributed to the reduction in hospital discharges, which might have otherwise increased, as occurred in children.

We conclude that transition to budesonide/formoterol maintenance and/or reliever therapy regimens in clinical practice can be achieved within a population; however, a degree of coprescribing of a SABA reliever may occur. Although we recognize that a causal effect cannot be assumed solely because of temporal associations, we can state that our observations that the transition to budesonide/formoterol prescribing was associated with reduced population asthma hospital admission risk are consistent with clinical trial findings of reduced severe exacerbation risk with ICS/formoterol reliever alone or maintenance and reliever therapy versus with SABA reliever-based regimens.^17^, ^18^, ^19^, ^20^

**Clinical implications**

The widespread transition to budesonide/formoterol maintenance and/or reliever regimens for asthma can be achieved in clinical practice and may be associated with a reduction in asthma hospital admissions.

## Disclosure statement

There was no funding for this study. The Medical Research Institute of New Zealand receives Independent Research Organization funding from the 10.13039/501100001505Health Research Council of New Zealand.

Disclosure of potential conflict of interest: R. Beasley has received institutional research funding from 10.13039/100004325AstraZeneca, 10.13039/501100001515Cure Kids (New Zealand), Genentech, and the 10.13039/501100001505Health Research Council of New Zealand, as well as personal fees from 10.13039/100004325AstraZeneca, Avillion, Teva Pharmaceuticals, and Cipla outside the submitted work; in addition, he is chair of the 10.13039/501100000988Asthma and Respiratory Foundation of New Zealand Adolescent and Adult Asthma Guidelines Group. The rest of the authors declare that they have no relevant conflicts of interest.

Data sharing statement: Data will be available to researchers who provide a methodologically sound proposal that has been approved by the study steering committee to achieve the aims outlined in the approved proposal. Data can be obtained through a signed data access agreement. The agreement can be obtained by e-mailing the principal investigator at the address richard.beasley@mrinz.ac.nz.

## References

[1] Beasley R., Beckert L., Fingleton J., Hancox R.J., Harwood M., Hurst M. (2020). Asthma and Respiratory Foundation NZ adolescent and adult asthma guidelines 2020: a quick reference guide. NZ Med J.

[2] Hatter L., Eathorne A., Hills T., Bruce P., Houghton C., Weatherall M., Beasley R. (2023). Patterns of asthma medication use in New Zealand after publication of national asthma guidelines. J Allergy Clin Immunol Pract.

[3] Huang Q.S., Wood T., Jelley L., Jennings T., Jefferies S., Daniells K. (2021). Impact of the COVID-19 nonpharmaceutical interventions on influenza and other respiratory viral infections in New Zealand. Nat Commun.

[4] Hills T., Kearns N., Kearns C., Beasley R. (2020). Influenza control during the COVID-19 pandemic. Lancet.

[5] Kung S., Hills T., Kearns N., Beasley R. (2023). New Zealand’s COVID-19 elimination strategy and mortality patterns. Lancet.

[6] Metcalfe S., Laking G., Arnold J. (2013). Variation in the use of medicines by ethnicity during 2006/07 in New Zealand: a preliminary analysis. NZ Med J.

[7] Statistics New Zealand Estimated resident population by age and sex (1991+) (Qrtly-Mar/Jun/Sep/Dec). https://infoshare.stats.govt.nz/SelectVariables.aspx?pxID=c7a1e00b-5e52-4c1f-b197-f35999d102c0.

[8] Global Initiative for Asthma (GINA) Global strategy for asthma management and prevention. https://ginasthma.org/wp-content/uploads/2019/06/GINA-2019-main-report-June-2019-wms.pdf.

[9] Beasley R., Holliday M., Reddel H.K., Braithwaite I., Ebmeier S., Hancox R.J. (2019). Controlled trial of budesonide-formoterol as needed for mild asthma. N Engl J Med.

[10] The New Zealand Medicines and Medical Devices Safety Authority (Medsafe). 2020. New Zealand Data Sheet: Symbicort Turbuhaler. https://www.medsafe.govt.nz/profs/datasheet/s/symbicortinh.pdf.

[11] Hardy J., Baggott C., Fingleton J., Reddel H.K., Hancox R.J., Harwood M., on behalf of the PRACTICAL Study Team (2019). Budesonide-formoterol reliever therapy versus maintenance budesonide plus terbutaline reliever therapy in adults with mild to moderate asthma (PRACTICAL): a 52-week, open-label multicentre, superiority, randomised controlled trial. Lancet.

[12] Hills T., Hatter L., Kearns N., Bruce P., Beasley R. (2021). COVID-19 border controls prevent a 2021 seasonal influenza epidemic in New Zealand. Public Health.

[13] Influenza and other viral respiratory surveillance dashboard ESR, New Zealand. https://www.esr.cri.nz/our-research/nga-kete/infectious-disease-intelligence/influenza-and-respiratory-surveillance/.

[14] Hatter L., Eathorne A., Hills T., Bruce P., Beasley R. (2021). Respiratory syncytial virus: paying the immunity debt with interest. Lancet Child Adolesc Health.

[15] Johnston S.L., Pattemore P.K., Sanderson G., Smith S., Lampe F., Josephs L. (1995). Community study of role of viral infections in exacerbations of asthma in 9-11 year old children. BMJ.

[16] Nicholson K.G., Kent J., Ireland D.C. (1993). Respiratory viruses and exacerbations of asthma in adults. BMJ.

[17] Sobieraj D.M., Weeda E.R., Nguyen E., Coleman C.I., White C.M., Lazarus S.C. (2018). Association of inhaled corticosteroids and long-acting β-agonists as controller and quick relief therapy with exacerbations and symptom control in persistent asthma a systematic review and meta-analysis. JAMA.

[18] Crossingham I., Turner S., Ramakrishnan S., Hynes G., Gowell M., Yasmin F. (2021). Combination fixed-dose beta agonist and steroid inhaler as required for adults or children with mild asthma. Cochrane Database Syst Rev.

[19] Beasley R., Harrison T., Peterson S., Gustafson P., Hamblin A., Bengtsson T. (2022). Evaluation of budesonide-formoterol for maintenance and reliever therapy among patients with poorly controlled asthma. A systematic review and meta-analysis. JAMA Netw Open.

[20] Beasley R., Bruce P., Houghton C., Hatter L. (2023). The ICS/formoterol reliever therapy regimen in asthma: a review. J Allergy Clin Immunol Pract.

