# Supplementary material for: Patterns of asthma medication use and hospital discharges in New Zealand
Source: J Allergy Clin Immunol Glob. 2024 Apr 10;3(3):100258. doi: 10.1016/j.jacig.2024.100258 (PMC11090902; doi:10.1016/j.jacig.2024.100258)

**ONLINE SUPPLEMENT**

**PATTERNS OF ASTHMA MEDICATION USE AND HOSPITAL DISCHARGES**

**IN NEW ZEALAND**

^1,2^Jonathan Noble MBBS, ^1,2^Lee Hatter MBBS, ^1^Allie Eathorne BSc, ^1^Thomas Hills DPhil,

^1^Orlagh Bean MBBS, ^1^Pepa Bruce MBBS, ^3^Mark Weatherall FRACP, ^1,2^Richard Beasley DSc

**Results:**

The trends in dispensing of budesonide/formoterol differed in a number of respects across the 12 to 34, 35 to 49, 50 to 64, and 65+ age groups. **[Figure S1; Table S1].** In the 12 to 34- and 35 to 49-year age groups, the budesonide/formoterol dispensing increased markedly from 2019, with a reduction in ICS, ‘other ICS/LABA’ and SABA dispensing during this period. In the 50 to 64 and 65+ age groups, there was a marked increase in budesonide/formoterol since 2019, together with a reduction in ICS, but minimal change in ‘other ICS/LABA’ and SABA dispensing. The overall dispensing rates were higher across all medication classes in the 65+ year age group, particularly the ‘other ICS/LABA’ and SABA dispensing. In the 12 to 34-year age group the change in budesonide/formoterol, ICS, ‘other ICS/LABA’ and SABA was +161%, -24%, -14% and -12%, compared with +74%, -16%, -4% and -4% in the 65+ age group respectively **[Table S1].**

**Table S1: Difference in rate of dispensing of inhalers between the period from July to December 2019 and the period from July to December 2022 by medication class in the a) <12, b) 12 to 34, c) 35 to 49, d) 50 to 65 and e) 65+ age groups.**

**a) <12 years**

|  | Inhalers dispensed per 100,000 population | | | |
| --- | --- | --- | --- | --- |
| Inhaler type | Jul-Dec 2019 | Jul-Dec 2022 | Difference | % change |
| Budesonide-formoterol | 320.21 | 904.18 | 583.97 | 182.4 |
| ICS | 5075.83 | 5299.77 | 223.95 | 4.4 |
| ICS-LABA (other) | 1410.68 | 1082.35 | -328.33 | -23.3 |
| SABA | 11976.93 | 11828.01 | -148.92 | -1.2 |

**b) 12 to 34 years**

|  | Inhalers dispensed per 100,000 population | | | |
| --- | --- | --- | --- | --- |
| Inhaler type | Jul-Dec 2019 | Jul-Dec 2022 | Difference | % change |
| Budesonide-formoterol | 2250.80 | 5883.72 | 3632.92 | 161.4 |
| ICS | 2390.43 | 1821.26 | -569.18 | -23.8 |
| ICS-LABA (other) | 3558.68 | 3070.64 | -488.04 | -13.7 |
| SABA | 8488.62 | 7515.61 | -973.01 | -11.5 |

**c) 35 to 49 years**

|  | Inhalers dispensed per 100,000 population | | | |
| --- | --- | --- | --- | --- |
| Inhaler type | Jul-Dec 2019 | Jul-Dec 2022 | Difference | % change |
| Budesonide-formoterol | 4529.57 | 9813.66 | 5284.09 | 116.7 |
| ICS | 3286.85 | 2461.28 | -825.57 | -25.1 |
| ICS-LABA (other) | 6762.79 | 6137.64 | -625.15 | -9.2 |
| SABA | 12728.02 | 11531.48 | -1196.53 | -9.4 |

**d) 50 to 64 years**

|  | Inhalers dispensed per 100,000 population | | | |
| --- | --- | --- | --- | --- |
| Inhaler type | Jul-Dec 2019 | Jul-Dec 2022 | Difference | % change |
| Budesonide-formoterol | 6843.19 | 13993.39 | 7150.20 | 104.5 |
| ICS | 5463.60 | 4434.54 | -1029.06 | -18.8 |
| ICS-LABA (other) | 11607.22 | 11431.16 | -176.05 | -1.5 |
| SABA | 18043.38 | 17725.18 | -318.20 | -1.8 |

**e) 65+ years**

|  | Inhalers dispensed per 100,000 population | | | |
| --- | --- | --- | --- | --- |
| Inhaler type | Jul-Dec 2019 | Jul-Dec 2022 | Difference | % change |
| Budesonide-formoterol | 10632.14 | 18509.18 | 7877.04 | 74.1 |
| ICS | 9933.53 | 8394.85 | -1538.68 | -15.5 |
| ICS-LABA (other) | 22440.36 | 21545.62 | -894.74 | -4.0 |
| SABA | 26058.45 | 24944.28 | -1114.17 | -4.3 |

**Figure S1: LOESS plot of medication dispensing rates monthly from January 2010 to December 2022 in the a) 12 to 34, b) 35 to 49, c) 50 to 64, and d) 65+ age group .**

Grey vertical lines indicate the date of the publication of the 2019 GINA update (April 2019),^8^ publication of the results of the Novel START trial (May 2019),^9^ approval of the indication for prescription of budesonide/formoterol (BUD/FORM) 200/6 ug as a reliever alone in New Zealand (June 2019),^10^ publication of the results of the PRACTICAL trial (September 2019),^11^ and publication of the New Zealand adolescent and adult asthma guidelines (June 2020).^1^ Shaded bands represent the 95% confidence intervals.

**Figure S1**

1. **12 to 34 years**


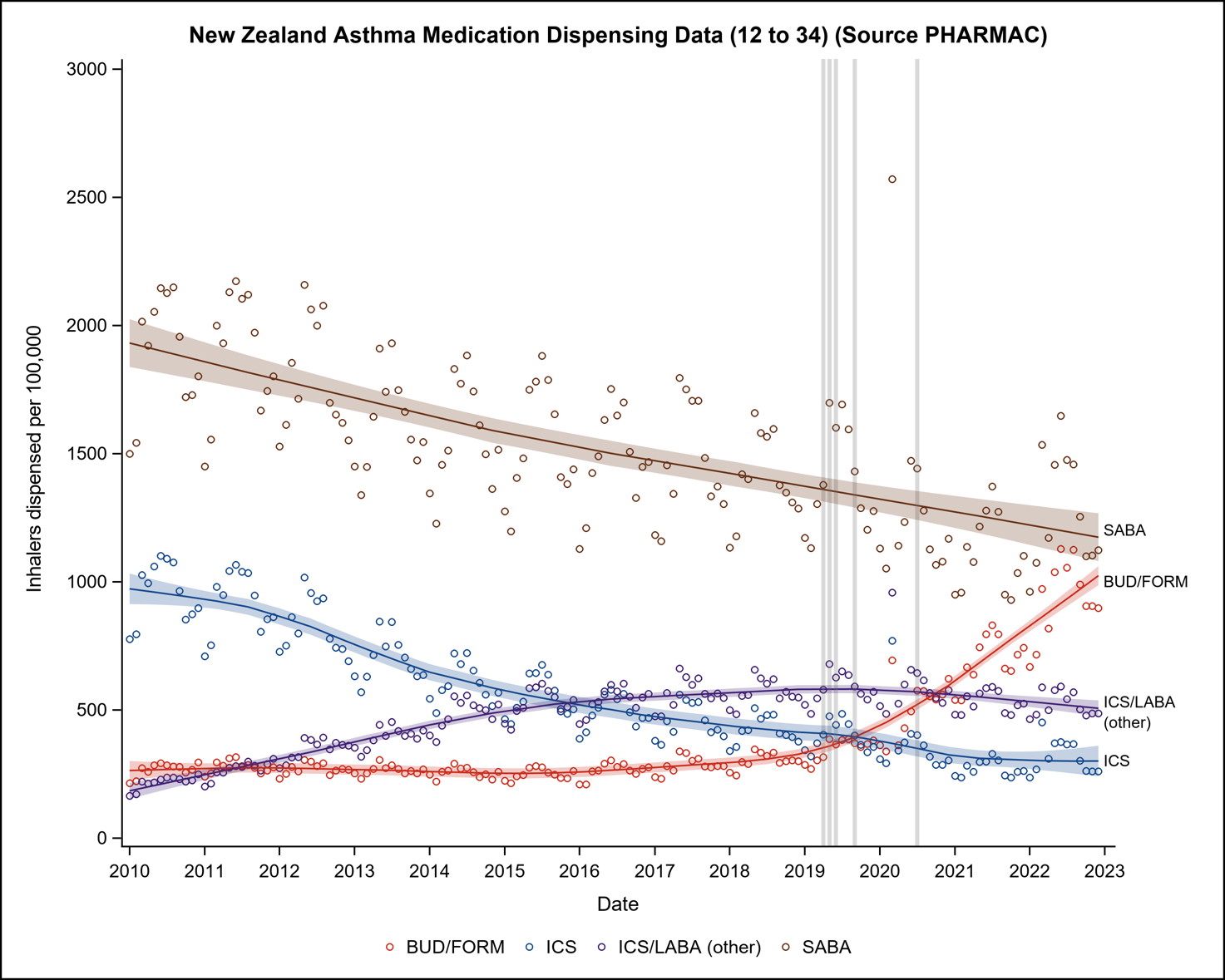


1. **35 to 49 years**

**
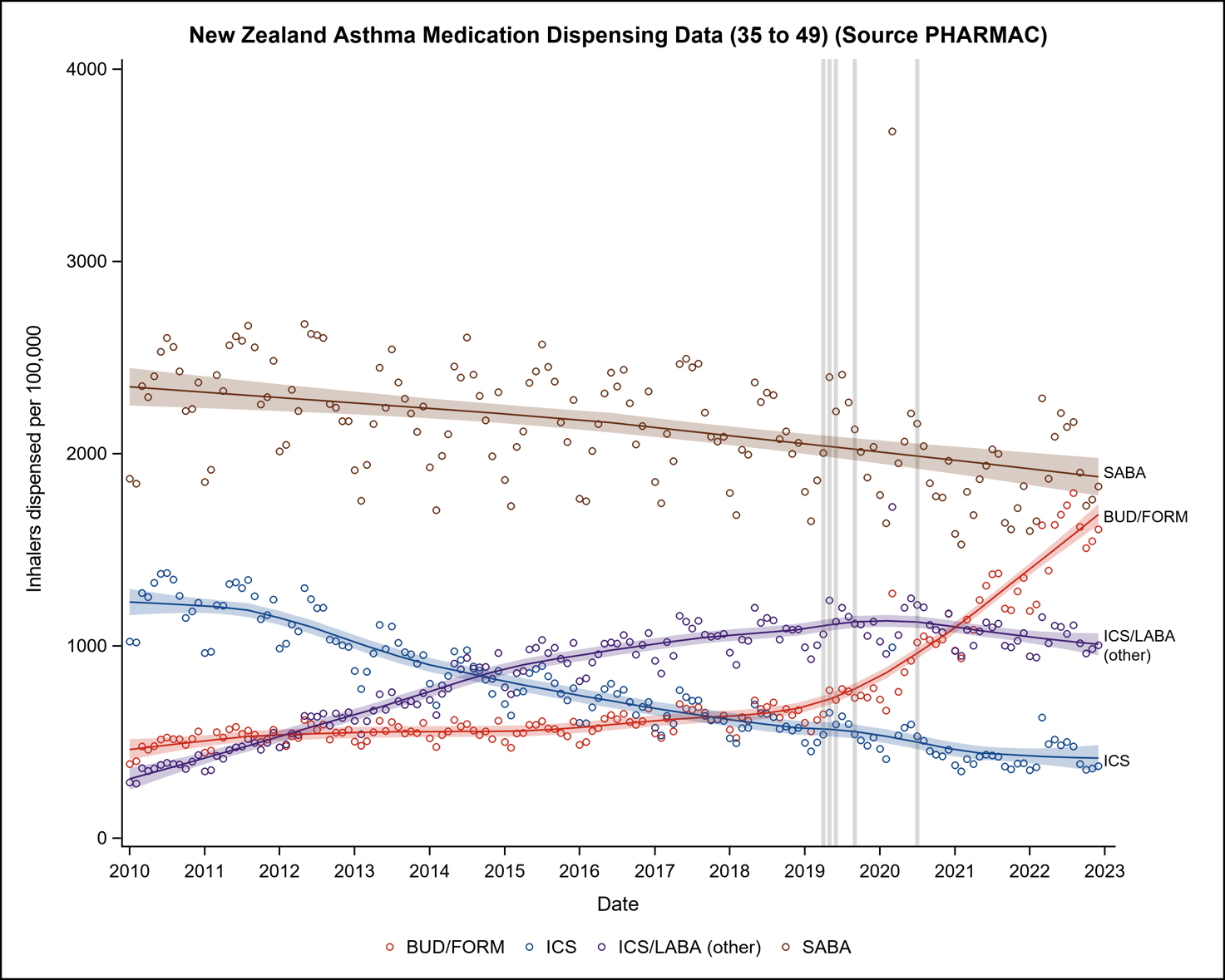
**

1. **50 to 64 years**

**
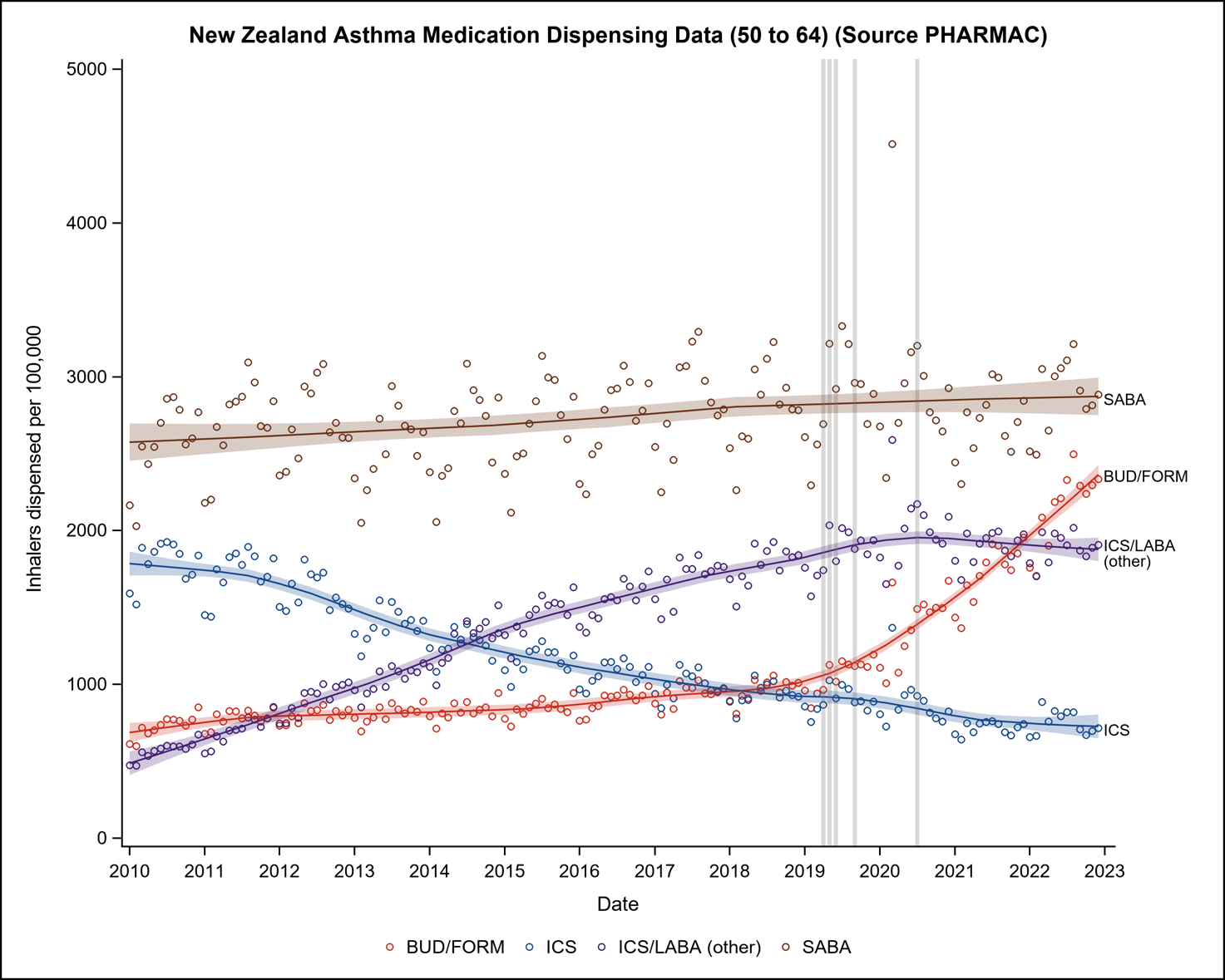
**

1. **65+ years**


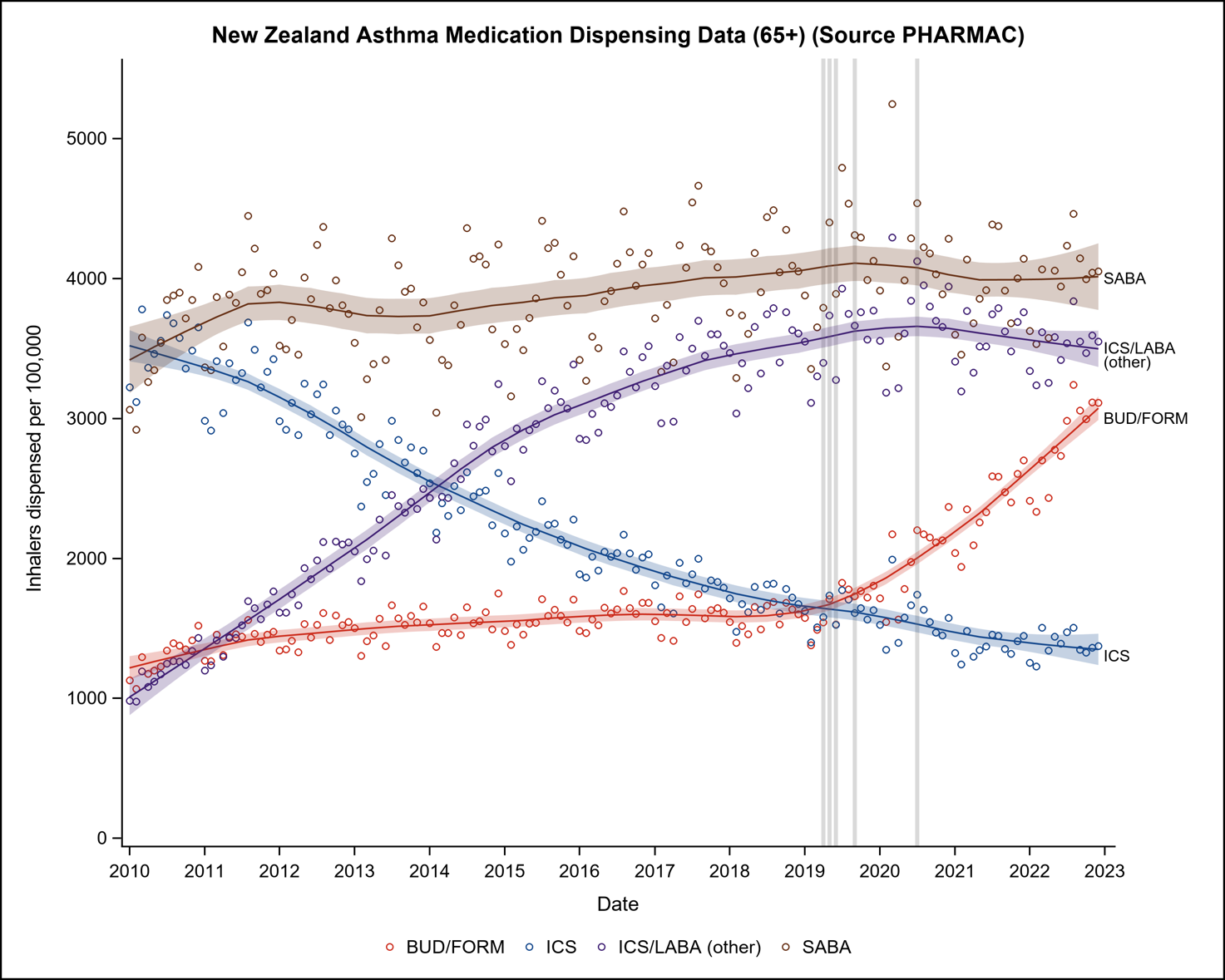

Supplement: Supplementary Fig and Table [file mmc1.docx]
